# Comprehensive Review of Mesothelioma Cases: From Diagnosis to Therapeutic Strategies

**DOI:** 10.7759/cureus.52859

**Published:** 2024-01-24

**Authors:** Abeer Qasim, Sai Vishnu Vardhan Allu, Patrik Schmidt, Harsh R Parikh, Sarah Moore, Laura Yapor, Maryam Soliman

**Affiliations:** 1 Internal Medicine, BronxCare Health System, New York City, USA; 2 Surgery, Regions Hospital, St. Paul, USA; 3 Orthopedic Surgery, University of Minnesota Medical School, Minneapolis, USA; 4 General Surgery/Internal Medicine, BronxCare Health System, New York City, USA; 5 School of Medicine, St. George's University, St. George's, GRD; 6 Medicine, American University of the Caribbean School of Medicine, Cupecoy, SXM; 7 Pulmonary and Critical Care, BronxCare Health System, New York City, USA; 8 Pulmonary and Critical Care Medicine, BronxCare Health System, New York City, USA

**Keywords:** pleural mesothelioma, biopsy, mesothelioma, malignant, epitheloid asbestos exposure

## Abstract

Mesothelioma is a rare and aggressive malignancy typically associated with asbestos exposure. We present the clinical and diagnostic journey of a 63-year-old male carpenter, who presented with concerning symptoms of shortness of breath and total right lung “white-out” on imaging. Comprehensive medical evaluation revealed the presence of malignant pleural mesothelioma. This study underscores the importance of considering mesothelioma as a potential diagnosis in individuals with occupational asbestos exposure and highlights patterns in diagnosing and managing this devastating disease. Early recognition and intervention are essential in improving outcomes for patients diagnosed with mesothelioma.

## Introduction

Mesothelioma, a formidable and elusive foe, continues to leave an indelible mark on the field of oncology. This rare and aggressive cancer, primarily associated with asbestos exposure, poses significant challenges in diagnosis, management, and patient outcomes. With its intriguing pathogenesis and devastating consequences, mesothelioma demands our unwavering attention [1]. The estimated yearly occurrence of mesothelioma in the United States is around 3,300 new cases yearly. The incidence of mesothelioma in the United States peaked around the year 2000 and is now declining, secondary to the control of asbestos exposure [2]. While the incidence varies across regions, occupational exposure to asbestos remains the leading risk factor, especially in mining, construction, and shipbuilding [3]. Prevalence rates highlight the lasting impact of asbestos use, with certain countries reporting higher mesothelioma rates due to past industrial practices and occupational hazards [4]. Tragically, mesothelioma carries a dire prognosis, with a high mortality rate attributed to its aggressive nature and late-stage diagnosis. The five-year survival rate for mesothelioma remains dishearteningly low, hovering around 10% [5]. Despite advancements in treatment options, the challenges posed by tumor heterogeneity, limited therapeutic efficacy, and resistance mechanisms underscore the need for further research and innovative strategies to improve patient outcomes. In this study, we present a captivating instance that offers unique insights into the multifaceted realm of mesothelioma. Through an in-depth analysis of the patient's clinical presentation, diagnostic workup, treatment modalities, and response to therapy, we aimed to contribute to the growing body of knowledge surrounding this complex malignancy. Diagnosing malignant mesothelioma is of paramount importance for various reasons. Early detection is crucial as symptoms often manifest in advanced stages, enabling more effective treatment interventions. Accurate diagnosis guides the formulation of an appropriate treatment plan, which may involve surgery, chemotherapy, radiation therapy, or a combination thereof. It also plays a key role in predicting prognosis and survival rates, providing individuals with valuable information about the expected course of the disease.

## Case presentation

The patient was a 63-year-old male who had been suffering from gradually worsening shortness of breath and dyspnea on exertion for a period of three months, which prompted him to visit his primary care doctor. He underwent a chest x-ray and subsequent CT chest outpatient, which revealed complete right-sided opacification. His primary care doctor (outside facility provider) then told the patient to visit his nearest hospital’s emergency room for further evaluation.

He presented to our emergency room feeling short of breath, but not in any acute distress. He was laying back in his chair without any significant discomfort and was saturating 100% of O_2_ on room air. He denied any fever and chills, any recent illnesses or exposure to illness, headaches, hemoptysis, chest pain or palpitations, diaphoresis, peripheral swelling or edema, vomiting, diarrhea, constipation, muscle or joint pain, polyuria, dysuria, or hematuria. He stated that more recently he has been coughing up white sputum without any blood, lost 10 pounds in the last three months, and felt more fatigued after working than normal. He is originally from Mexico and moved to the United States over 20 years ago, and works as a carpenter in the inner city. He has a past medical history of hypertension for which he takes metoprolol 50 mg once daily, and asthma for which he only has an albuterol rescue inhaler at home that he uses as needed. He denied any family past medical history. The patient's vital signs were within normal ranges, with a body temperature of 36.5°C, a pulse rate of 91 beats per minute, a respiratory rate of 18 breaths per minute, a systolic blood pressure of 128 mmHg, a diastolic blood pressure of 87 mmHg, and a 100% oxygen saturation level on room air. His initial set of labs is shown in Table 1.

**Table 1:** Initial lab values of the patient.

Result	Value	Range
White blood cell count	8.7 k/µL	4.8-10.8 k/µL
Hemoglobin	15.5 g/dL	12.0-16.0 g/dL
Platelet	206 k/µL	150-400 k/µL
Neutrophil %	66.3%	40.0-70.0%
Sodium (serum)	140 mEq/L	135-145 mEq/L
Potassium (serum)	5 mEq/L	3.5-5.0 mEq/L
Blood urea nitrogen (serum)	13 mg/dL	8.0-26.0 mg/dL
Creatinine (serum)	0.6 mg/dL	0.5-1.5 mg/dL
Partial thromboplastin time (PTT)	35.7 s	27.2-39.6 s
Prothrombin time	12.2 s	9.9-13.3 s
International normalized ratio (INR)	1.04	0.85-1.14
Lactic acid level	1.3 mmol/L	0.5-1.6 mmol/L

**Figure 1:**
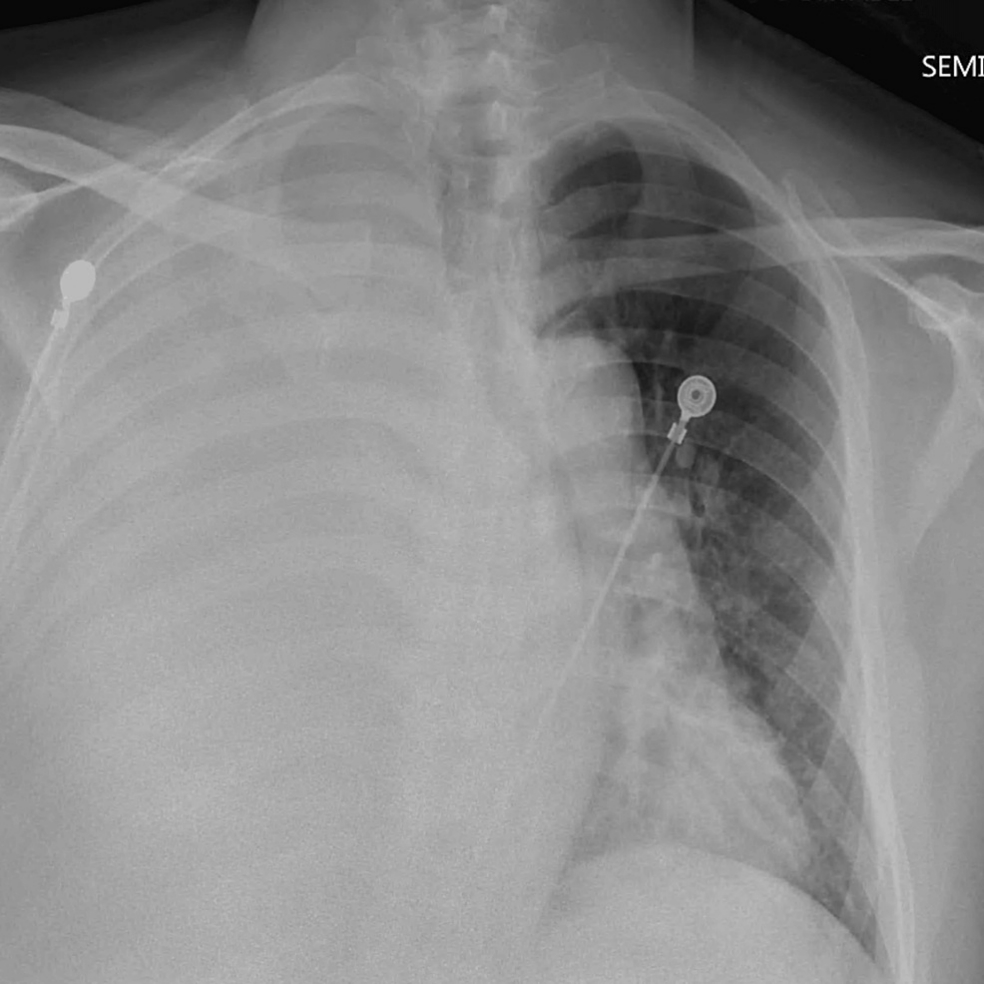
Chest x-ray showed a large right pleural effusion.

His chest x-ray showed a large right pleural effusion, with a follow-up CT scan of the chest showing the same, along with nodularity of the lung parenchyma concerning neoplasm and a possible obstructive endobronchial lesion. It also showed a mediastinal shift towards the left hemithorax. The patient was started on broad-spectrum antibiotic coverage with vancomycin, Zosyn, and azithromycin. Chest x-ray on the day of presentation is shown in Figure 1.

**Figure 2:**
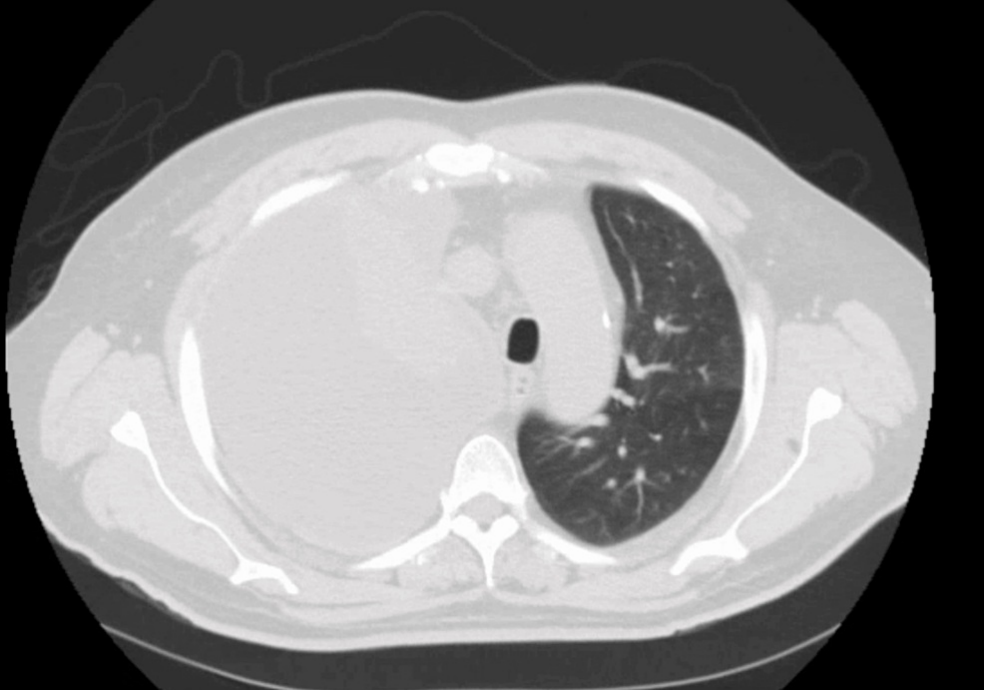
CT chest showing large right pleural effusion.

**Figure 3:**
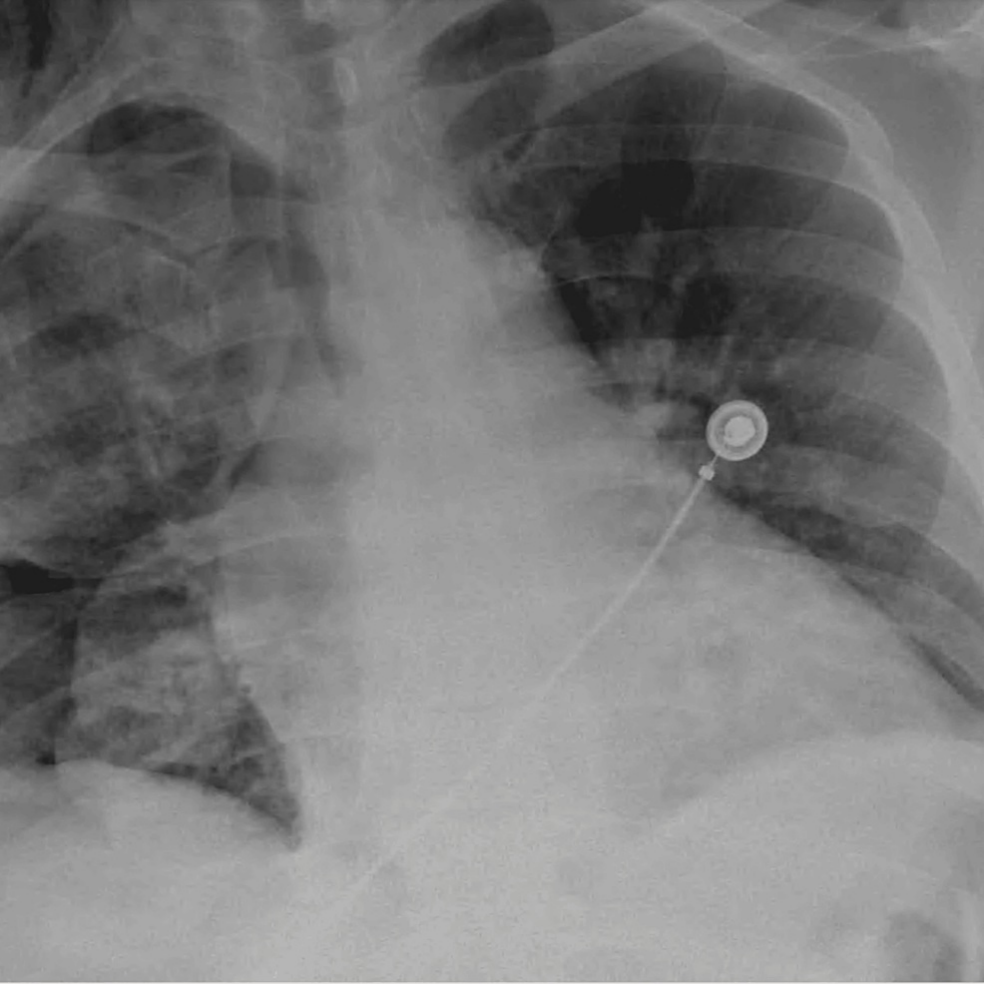
Chest x-ray post thoracocentesis.

CT scan revealed a large right pleural effusion (Figure 2). A repeat chest x-ray was done after thoracocentesis (Figure 3). He underwent thoracentesis of the right-sided collection which showed an exudative effusion. The fluid characteristics of thoracocentesis are shown in Table 2.

**Table 2:** Thoracocentesis fluid findings. ADA: adenosine deaminase

Color	Yellow (normal:clear)
Appearance	Hazy (normal straw color)
White blood cells	340 cells/mm^3^ (normal: <1000 WBC/µL)
Red blood cells	1775 million cells/mm^3^ (normal: <100 RBCs/µL)
Segment count	28% (normal 5%)
Lymphocyte count	62% (normal 2-30% lymphocytes)
Eosinophil count	10% (normal <10%)
Albumin	3.4 g/L (9-45 g/L)
Amylase	41 U/mL (<250 U/mL)
Cholesterol	74 mg/dL (<50 mg/dL)
Glucose	73 mg/dL (70-120 mg/dL)
Lactate dehydrogenase	175 (<50% plasma concentration)
Protein	5 g/dL (<2% {1-2 g/dL})
ADA	18.4 U/L (<9.2 U/L)

**Figure 4:**
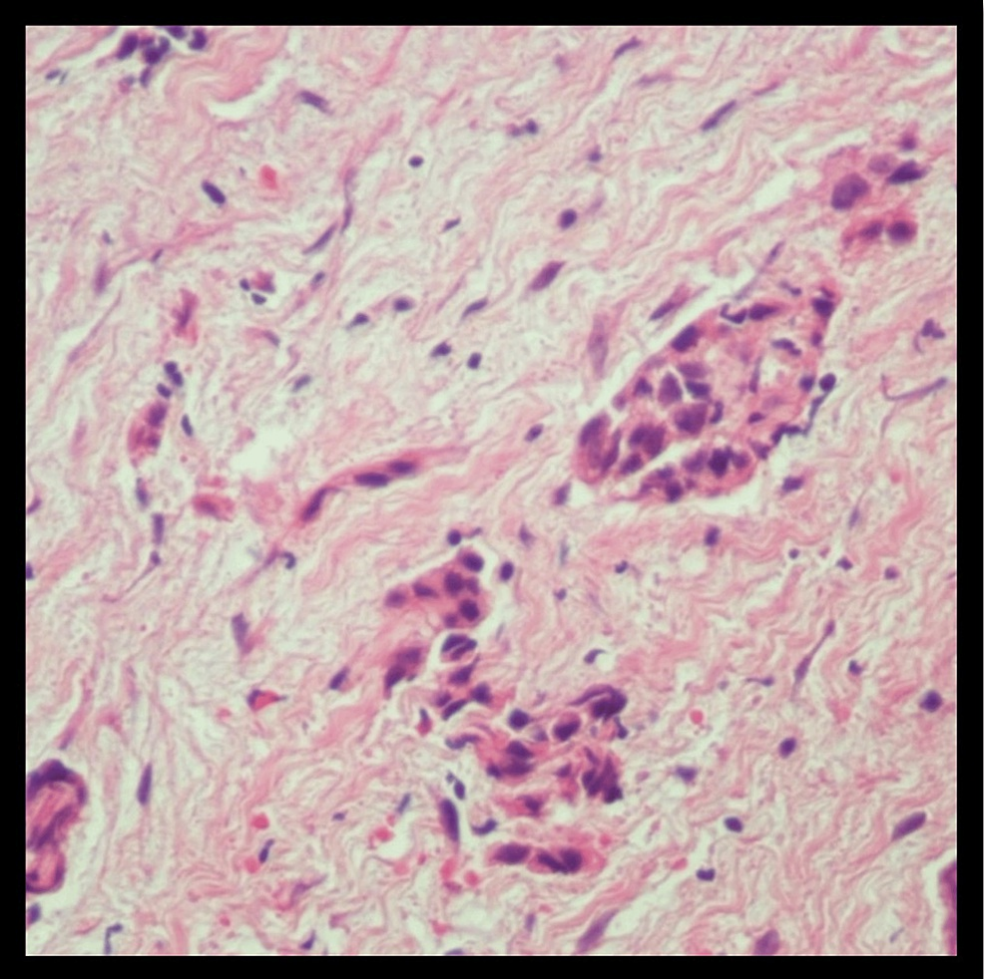
Biopsy showed sections demonstrating fibroadipose tissue, partially cauterized (magnification x40).

**Figure 5:**
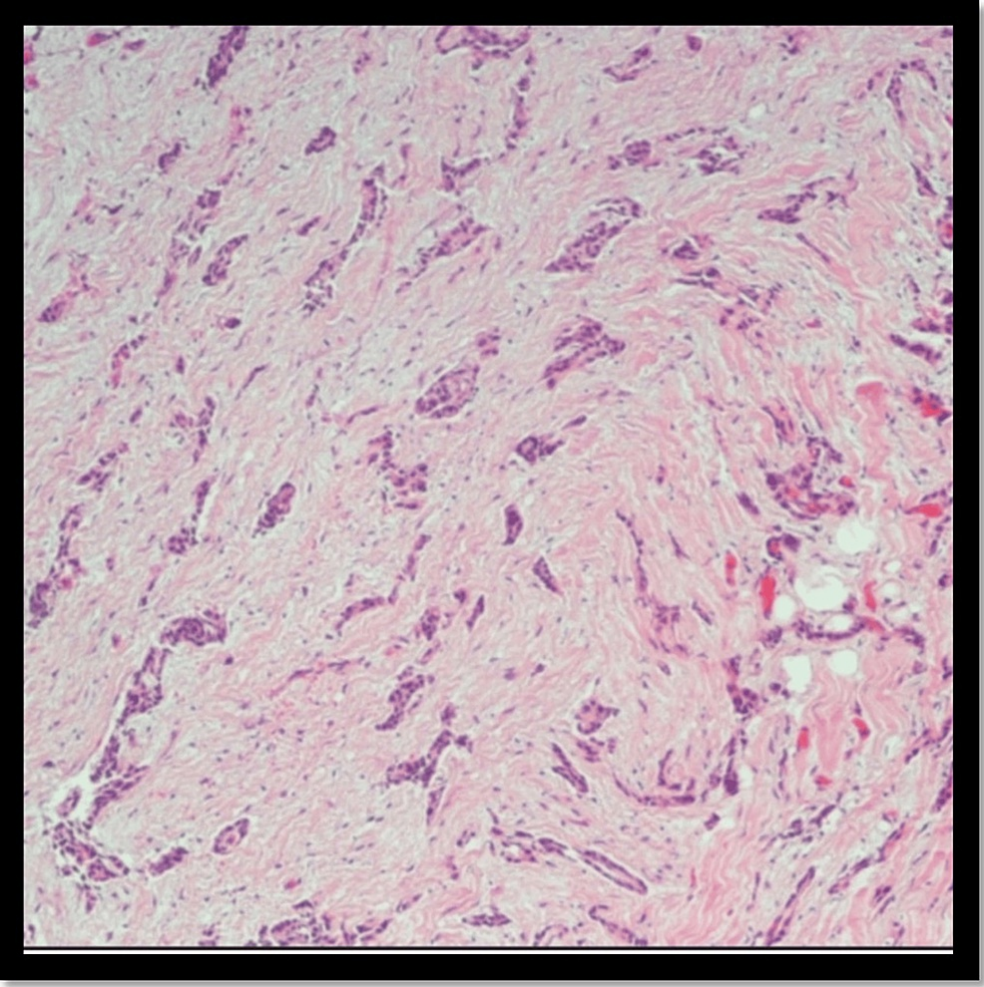
Biopsy showed infiltrating cords, tubules, and nests of neoplastic cells with low nuclear grade and no necrosis (magnification x40).

Furthermore, his HIV screen was negative. He also underwent video-assisted thoracoscopic surgery (VATS) during which pleural biopsies were taken (Figures 4, 5). These came back positive for epithelioid mesothelioma, low-grade. Oncology was consulted because of these findings, and chemotherapy using cisplatin with pemetrexed, and bevacizumab is being planned for the patient.

## Discussion

Epithelioid mesothelioma is a malignant tumor arising from mesothelial cells of the pleura and pleural cavities. In the early stages, it presents as small nodules arising from the parietal pleura, with rare involvement of the visceral pleura [6,7]. With the progression of the disease, the nodules coalesce to form an extensive thickened-rigid tumor that can merge the parietal and visceral pleura, encasing the lung and restricting ventilatory capacity [8]. The clinical symptomatology of mesothelioma involves a gradual onset of nonspecific respiratory symptoms, including the following: chest pain, dyspnea, cough, and hoarseness [6,7]. Additionally, nonspecific systemic B-signs of neoplastic disease can also be present in patients with chronic disease as follows: weight loss, night sweats, and chronic fatigue [7-9]. In the acute setting, patients commonly present with physical examination and radiological findings consistent with pleural effusion, confirmed as exudative effusion with cytology following thoracentesis [6,7]. Other radiological findings can include pleural thickenings, nodules, or plaques [7,10]. With clinical suspicion of mesothelioma, a thorough patient interview should be conducted to inquire about potential hazardous exposure risks, primarily asbestos exposure [9]. The patient in this study presented with acute signs consistent with a unilateral pleural effusion and chronic signs of weight loss and chronic fatigue. Cytology following the patient’s thoracentesis identified the fluid to be exudative, most likely secondary to the patient's underlying malignancy. Additionally, a thorough review of the patient’s history also identified a potential long-term occupational exposure to asbestos. The combination of these clinical findings and potential hazardous exposure provided clinical evidence to support the clinical suspicion of mesothelioma. The histopathology of epithelioid mesothelioma involves stromal-myxoid features with heterogeneous architectural and cytological patterns [7,8]. However, the keystone for histopathological diagnosis of mesothelioma involves immunohistochemical (IHC) pathological analysis [11,12]. Pancytokeratin staining is the primary test in the IHC confirmation for mesothelioma, with a sensitivity >90% when differentiating mesothelioma from other cancers in the differential [7,11].

Furthermore, positive IHC-staining for cytokeratins 5/6/7/20 is specific to the epithelioid classification of mesothelioma, differentiating it from sarcomatoid or biphasic mesothelioma subtypes [11-13]. Additionally, IHC-staining for calretinin, Wilms tumor 1 (WT-1) protein-antigen, podoplanin (D2-40), thrombomodulin, mesothelin, methylthioadenosine (MTAP), and BRCA1-associated protein (BAP1) are also useful in the histopathological confirmation of epithelioid mesothelioma [12-14]. Relevant to this case, pathological investigation of the pleural cell biopsy identified nests of neoplastic cells with low nuclear-grade and architectural heterogeneity, infiltrating cords and tubules. IHC-staining was positive for cytokeratin-5, cytokeratin-7, calretinin, WT-1, and weak-staining for BAP1 and MTAP. The constellation of these pathological findings supported the final diagnosis of epithelioid mesothelioma for our patient. The etiopathogenesis of malignant mesothelioma is intricately linked to asbestos exposure, encompassing factors like duration, mode, and frequency of contact with asbestos. Prolonged exposure, particularly in occupational settings like construction, shipbuilding, and asbestos mining, heightens the risk, with individuals engaged in such professions over extended periods being more vulnerable. Additionally, nonoccupational exposure through secondary contact, where asbestos fibers are brought home by those working in related industries, contributes to the complexity. The frequency and intensity of exposure play a crucial role, impacting the likelihood of developing mesothelioma. Various types of asbestos fibers, including chrysotile, amosite, and crocidolite, pose risks, with crocidolite considered more potent. Environmental exposure, whether through residing in asbestos-rich areas or proximity to industries utilizing asbestos, adds another layer to the disease's etiology. The use of asbestos in products like insulation and roofing materials, particularly in occupational settings, significantly contributes to the risk. The long latency period of mesothelioma, with symptoms emerging decades after initial exposure, underscores the importance of considering historical exposure when assessing risk factors. Overall, a comprehensive understanding of these multifaceted aspects is essential for recognizing and addressing the complexities of malignant mesothelioma development.

Clinical management of mesothelioma involves a combination of acute management in presenting symptoms and chronic therapeutic treatment [6]. Initial management involves managing intrathoracic pulmonary symptoms, including dyspnea, cough, chest pain, and pleural effusion [6,8]. This can involve therapeutic thoracentesis, with diagnostic cytology or video-assisted thoracic surgery (VATS) for surgical resection, both for diagnostic biopsy and to improve ventilatory lung function [15,16]. Long-term therapeutic management of mesothelioma involves definitive surgical management, chemoradiation therapy, or combination management [15]. Definitive surgical management, unlike initial VATS-resection, involves a multimodality approach to remove all malignant neoplastic nests - pleurectomy with decortication and extrapleural pneumonectomy [10,15]. Chemoradiation therapy can involve directed radio beam radioactive therapy or directed chemotherapeutic treatment with cisplatin and neoadjuvant pemetrexed [17,18]. In the present case, the patient underwent initial therapeutic thoracentesis to improve ventilation, followed by VATS-guided surgical resection for biopsy and severity assessment. Definitive management consisted of combination therapy with pleurotomy surgical management with cisplatin-pemetrexed adjuvant chemotherapy.

Clinical prognosis is dependent on the clinically and pathologically graded severity of the malignancy. Epithelioid mesothelioma severity grading is based on a combination of nuclear atypia and necrosis involvement. Low-grade tumors involve those with either grade I nuclear atypia (mild atypia: <1 mitosis/2 mm^2^) or grade II nuclear atypia (intermediate atypia: 2-4 mitoses/2 mm^2^), without necrosis-involvement. Conversely, high-grade tumors involve any tumor with necrosis-involvement or grade III nuclear atypia (high atypia: >5 mitoses/2 mm^2^), with or without necrosis-involvement [7]. Clinically, the prognosis is dependent on classical tumor, node, metastasis (TNM) staging patterns and patient-performance characteristics including age >75 years, elevated lactate dehydrogenase (LDH), and overall hematological adaptability [6,8,15,19]. The pathological features of our patient suggested a low-grade epithelioid mesothelioma with no nodal involvement or metastasis. Clinically, our patient was otherwise young and healthy, with no other hematological disorders. In summary, the overall prognosis of our patient is positive due to good overall clinical health and low-grade pathological characteristics.

## Conclusions

In summary, mesothelioma, a malignancy primarily linked to asbestos exposure, requires a multifaceted approach for its diagnosis and treatment. This study has deepened our understanding of its pathogenesis, clinical presentation, and therapeutic options. Timely and accurate diagnosis is crucial, with advanced imaging techniques like PET-CT, MRI, and thoracoscopy aiding in disease assessment. Surgical interventions, including cytoreductive surgery and pleurectomy/decortication, are aimed at removing visible tumors and improving local disease control. Adjuvant therapies such as chemotherapy and radiation target residual microscopic disease. Novel treatments, like immunotherapy and targeted therapies, have advanced mesothelioma management. Immune checkpoint inhibitors like pembrolizumab and nivolumab enhance the immune system's cancer-fighting capabilities. Targeted therapies inhibiting specific signaling pathways show promise in specific genetic subgroups. Despite these advances, mesothelioma remains challenging due to its aggressiveness, limited treatment options, resistance mechanisms, and delayed diagnosis, resulting in a poor prognosis. A multidisciplinary approach involving various medical professionals is essential for personalized and comprehensive care. Ongoing research efforts focused on understanding mesothelioma's molecular mechanisms and identifying new therapeutic targets are crucial for improving patient outcomes and survival rates.
